# Modelling geographical accessibility to urban centres in Kenya in 2019

**DOI:** 10.1371/journal.pone.0251624

**Published:** 2021-05-14

**Authors:** Peter M. Macharia, Eda Mumo, Emelda A. Okiro

**Affiliations:** 1 Population Health Unit, Kenya Medical Research Institute-Wellcome Trust Research Programme, Nairobi, Kenya; 2 Centre for Tropical Medicine and Global Health, Nuffield Department of Medicine, University of Oxford, Oxford, United Kingdom; University of Liverpool, KENYA

## Abstract

**Background:**

Access to major services, often located in urban centres, is key to the realisation of numerous Sustainable Development Goals (SDGs). In Kenya, there are no up-to-date and localised estimates of spatial access to urban centres. We estimate the travel time to urban centres and identify marginalised populations for prioritisation and targeting.

**Methods:**

Urban centres were mapped from the 2019 Kenya population census and combined with spatial databases of road networks, elevation, land use and travel barriers within a cost-friction algorithm to compute travel time. Seven travel scenarios were considered: i) walking only (least optimistic), ii) bicycle only, iii) motorcycle only, iv) vehicle only (most optimistic), v) walking followed by motorcycle transport, vi) walking followed by vehicle transport, and vii) walking followed by motorcycle and then vehicle transport (most pragmatic). Mean travel time, and proportion of the population within 1-hour and 2-hours of the urban centres were summarized at sub-national units (counties) used for devolved planning. Inequities were explored and correlations between the proportion of the population within 1-hour of an urban centre and ten SDG indicators were computed.

**Results:**

A total of 307 urban centres were digitised. Nationally, the mean travel time was 4.5-hours for the walking-only scenario, 1.0-hours for the vehicle only (most optimistic) scenario and 1.5-hours for the walking-motorcycle-vehicle (most pragmatic) scenario. Forty-five per cent (21.3 million people) and 87% (41.6 million people) of Kenya’s population resided within 1-hour of the nearest urban centre for the least optimistic and most pragmatic scenarios respectively. Over 3.2 million people were considered marginalised or living outside the 2-hour threshold in the pragmatic scenario, 16.0 million Kenyans for walking only, and 2.2 million for the most optimistic scenario. County-level spatial access was highly heterogeneous ranging between 8%-100% and 32%-100% of people within the 1-hour threshold for the least and most optimistic scenarios, respectively. Counties in northern and eastern parts of Kenya were generally most marginalised. The correlation coefficients for nine SDG indicators ranged between 0.45 to 0.78 and were statistically significant.

**Conclusion:**

Travel time to urban centres in Kenya is heterogeneous. Therefore, marginalised populations should be prioritised during resource allocation and policies should be formulated to enhance equitable access to public services and opportunities in urban areas.

## Introduction

Key to the realisation of most Sustainable Development Goals (SDGs) is physical access to healthcare, education and financial services, trading centres, employment opportunities and essential government services [1–4]. The providers of these services are often concentrated in urban centres [5–7]. Thus, spatial accessibility to urban centres can be used to evaluate how easily populations can access such services and their likely involvement in activities and services domiciled in urban centres [2, 4, 8, 9].

Kenyan urban centres are major educational hubs where literacy rates and school completion rates (e.g., primary school education) are higher. Moreover, most universities in Kenya are situated in urban centres [10–12]. Compared to rural areas, urban centres are often the main beneficiaries of large scale development investments such as airports, superhighways and power grids that lead to better transport and communication connectivity [11]. Further, urban areas serve as trading hubs for agricultural products and thus tend to have better food security [13, 14]. Urban centres host many financial institutions such as banks and micro finance institutions where small business loans can be accessed. Major business headquarters, and important government services and parastatals are predominantly located in large urban centres [2, 12, 15]. Therefore, compared to rural areas, employment opportunities are higher in urban areas and, as a result, people tend to migrate from rural to urban areas in search of employment opportunities and better living conditions [16].

At the aggregate level, child survival and other health outcomes are better in urban areas than in rural areas. Other urban advantages include better access to water and sanitation, birth registration, access to healthcare services, housing, and lower rates of stunting and under-five mortality [11, 17, 18]. Consequently, spatial accessibility to urban areas is routinely used as a predictor of population health and other associated positive outcomes [19–23]. A better understanding of the heterogeneities in spatial access to urban areas in Kenya will facilitate the identification of the populations that are marginalised from key services and institutions for more targeted action. This is enshrined within the SDGs’ fundamental principle of *leaving no one behind and with a focus on reaching those who are most marginalised*, *first* [24–26].

According to the 2010 Kenyan Constitution [27], a marginalized community is one that has been unable to fully participate in the integrated social and economic life of Kenya as a whole because of its relatively small population or for any other reason. The Kenya government through the Commission on Revenue Allocation (CRA) has identified marginalized areas in order to facilitate better resource allocation in the country [28]. CRA considers marginalised areas, as areas cut off from national growth mainly due to distance and inaccessibility [28]. These deprived areas are characterized by poor road networks, lack of access to clean water and improved sanitation, insufficient electricity, and insecurity leading to limited economic growth [28]. In this paper, we define marginalization from services as living outside a 2-hour travel catchment of the nearest urban area [28–31] even though there are no formally defined thresholds for access to urban areas. Nevertheless, we used 1-hour and 2-hour cut-offs because they are routinely used in spatial healthcare access analyses [30–35].

Despite the opportunities available in urban areas, there are some negative outcomes associated with urbanization. The increased population pressure in urban areas often leads to road congestion, vehicle induced injuries, and substantial air and industrial pollution resulting in a higher prevalence of respiratory diseases [36, 37]. The rapid growth of the world’s urban population, especially in the global south, is also associated with inadequate urban planning and overstretched public services. Due to urban sprawl, there is also an increase in the incidence of slums that are associated with overcrowding, inadequate water and sanitation, poor housing and living conditions, and limited education and healthcare provision [17, 38].

Because of the importance of urban areas in modern socioeconomic development, there have been several efforts to map physical access to urban areas across the globe. For instance, a global accessibility gridded surface to major cities of at least 50,000 people in the year 2000 [39] was created and later updated in 2015 to reflect better data availability and mapping methods [2]. The 2015 version was further improved to capture access to nine classes of different sized urban areas based on population differentials from 5,000 to five million people [4]. Yet, these global accessibility gridded surfaces mainly relied on global datasets which did not capture local definitions of urban centres [4, 39, 40]. The common lower limit of 5,000 inhabitants in an urban area [4] is arbitrary since many countries use different population cut-offs [10, 19, 41] and access to smaller urban areas is equally important [4]. Where local definitions of urban centres have been used [15, 16], different and alternative travel scenarios used within a country [42] have not always been considered. The use of various travelling scenarios presents different alternatives for accessing an urban centre and provides insights into the importance of transport modes [8]. For example, the exponential increase in the use of motorcycles in Kenya as a means of transportation has significant implications on access to urban areas [43].

These two drawbacks in the global surfaces limit the contextualization and applicability of existing gridded surfaces to economic planning within countries such as Kenya. Further, the latest gridded surfaces date to 2015; yet there is a high likelihood of many changes in the number and spatial extent of urban centres across the globe that necessitates an update of the 2015 grids. Moreover, there is a need to account for more recent localized urbanization trends. For these reasons, we extend the 2015 gridded surface study by providing an updated and localised accessibility surface to urban centres at 1 x 1 km spatial resolution for Kenya for 2019. Updated maps of urban centres, road networks, land use, elevation and travel barriers were combined with seven different locally adapted travel scenarios in a geospatial framework to compute travel time to the nearest urban centre. Spatial access variations for populations within 1-hour and 2-hours of travel time per county (which is Kenya’s planning unit [27, 44]) were explored and correlated with ten SDG indicators.

## Kenya context

### Population distribution and urbanization

**Fig 1:**
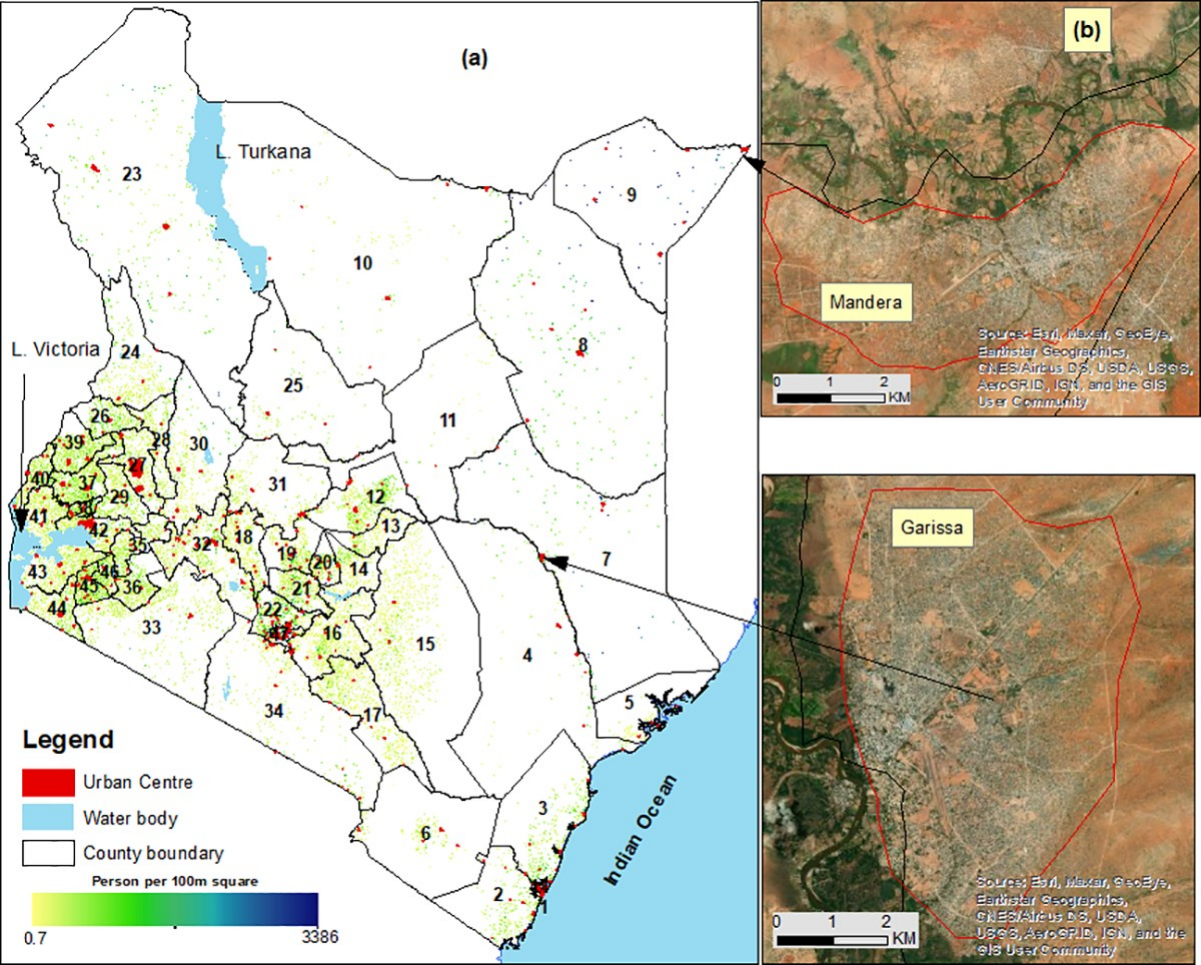
**A)** Urban centres in Kenya based on 2019 Kenya population census overlaid on Kenya counties (numbered-see footnote) and water bodies (blue). **B)** Inset shows two zoomed-in urban areas namely Garissa and Mandera in North-eastern Kenya. Source: authors. Mombasa [1], Kwale [2], Kilifi [3], Tana River [4], Lamu [5], Taita Taveta [6], Garissa [7], Wajir [8], Mandera [9], Marsabit [10], Isiolo [11], Meru [12], Tharaka-Nithi [13], Embu [14], Kitui [15], Machakos [16], Makueni [17], Nyandarua [18], Nyeri [19], Kirinyaga [20], Murang’a [21], Kiambu [22], Turkana [23], West Pokot [24], Samburu [25], Trans Nzoia [26], Uasin Gishu [27], Elgeyo-Marakwet [28], Nandi [29], Baringo[30], Laikipia [31], Nakuru [32], Narok [33], Kajiado [34], Kericho [35], Bomet [36], Kakamega [37], Vihiga [38], Bungoma [39], Busia [40], Siaya [41], Kisumu [42], Homa Bay [43], Migori [44], Kisii [45], Nyamira [46], Nairobi [47].

Kenya’s population was 47.6 million in 2019 with an inter-censual growth rate of 2.2% [45]. The average population density was 82 per square kilometer (sq.km) and was highly variable across the 47 counties ranging between less than 20 people per sq.km to over 500 people per sq.km [45] (Fig 1). Population distribution in Kenya is largely driven by availability of resources, climate, agricultural lands, soil types and rainfall [46]. Majority (over 80%) of the population is concentrated in approximately 20% of the country’s arable land where agriculture is predominant. Nine sparsely populated (15 persons per sq.km) arid counties in Northern Kenya [47] occupy large geographical extents, over 62% of Kenya’s land area, and are mainly inhabited by pastoralists who account for only 12% of Kenya’s population [45, 47, 48]. Large swaths of Kenya’s unpopulated land mass are characterized by large conservation areas and deserts. Conversely, 18 counties located in the Lake Victoria and Central regions and partly along the Indian ocean, covering 22% of Kenya’s land area, account for over 60% of the country’s total population [45]. The over-dispersion of people in the country’s arid counties has consequences on the distribution of resources, planning, spatial distribution of urban centres and their physical accessibility.

**Fig 2:**
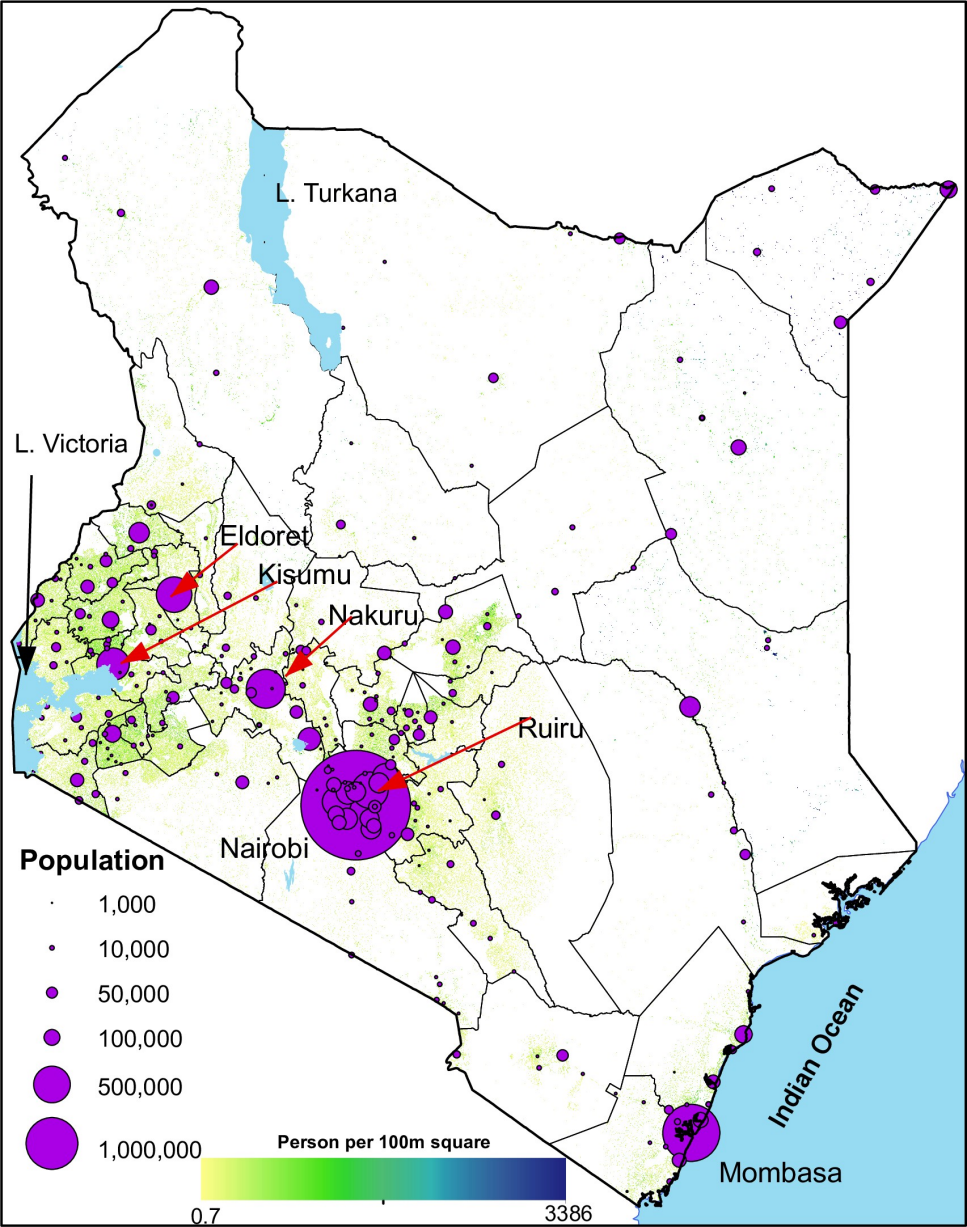
The relative population size of each urban area. Nairobi, Mombasa, Nakuru, Eldoret, Kisumu, and Ruiru, the most populous urban areas have been labelled. Population count constrained to areas with settlements per 100m square is shown ranging from low (yellow) to high (blue) [51]. Source: authors.

Approximately, one-third (31%) of Kenya’s population (14.8 million people) in 2019 was enumerated in urban areas. Kenya has a primate urban landscape [49] with Nairobi being three times larger than the second largest urban centre (Figs 1 and 2). Consequently, eight counties namely; Mombasa, Nairobi, Kisumu, Machakos, Kiambu, Uasin Gishu, Nakuru, and Kajiado account for 70% of the urban population. In these “*urban counties*”, 68 people out of 100 live in an urban area whereas only 23 out of 100 people live in an urban area in the nine arid counties that constitute only 8.3% of Kenya’s urban population [45]. These patterns have been observed historically [50].

Larger urban areas have better access to services compared to the smaller centres [49]. There has been a significant increase in the number of urban areas in Kenya from 215 in 2009 to 307 in 2019. This 43% increase is mainly due to rural-to-urban migration and natural urban increase [49, 52]. In 2009, only 14 urban centres had a population of at least 100,000 compared to 22 in 2019, an increase of 57 percent. Among the ten fastest-urbanizing areas in Kenya, three were satellite towns (Thika, Juja, and Kitengela) within the Nairobi metropolitan area; with similar patterns observed in other metropolitan areas [49]. The population and economic growth of Nairobi and its satellite towns has been driven by improved infrastructure and increasing cost of living within city limits [49]. Similar to the country’s overall population distribution, the spatial distribution of Kenya’s urban centres and their corresponding population is largely driven by the availability of resources, agricultural lands, and rainfall [46].

In the last decade, there has been a reduction in the rural-urban divide in Kenya through devolution and the attendant expansion of services to rural areas. For example, the need for personal banking has substantially declined after the introduction of *MPESA* or mobile money agents across the country. There are over 160,000 *MPESA* (mobile money—under one telecommunication company) agents distributed across Kenya. Over 20 million Kenyans own a mobile phone that can be used to engage with e-services such as mobile payments and delivery services through motorcycles. Majority of national government services (e.g., renewal of driving license, application of police clearance certificate and travel passport) are now offered via its online E-citizen portal. This has reduced trips to urban areas for some government services for those with access to internet services. Substantial investment in rural electrification in recent years has also reduced rural-urban quality of life disparities in Kenya [53]. Despite these developments, there are still many services that are domiciled within the urban areas.

### Transportation in Kenya

**Fig 3:**
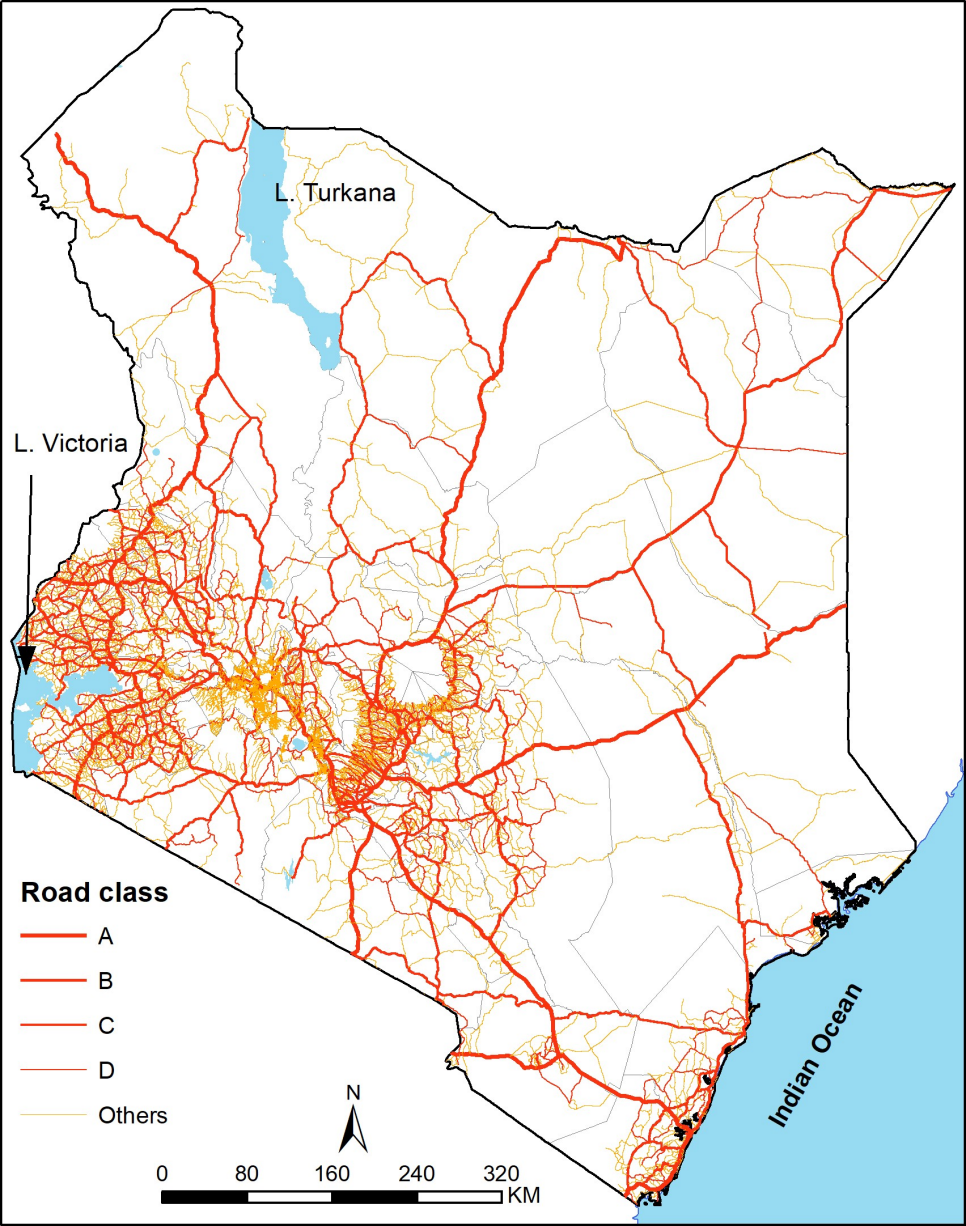
A map of road network in Kenya. Source: authors.

The transport network in Kenya consists of road, rail, maritime and inland water, and air. The system contributes about 8% of the country’s Gross Domestic Product [54, 55]. Access to urban areas is mainly through the road network which accounts for approximately 93% of all cargo and passenger traffic in the country [54]. It is about 177,800 km long with approximately 15% of it being paved [54, 56]. The last decade saw the construction, rehabilitation and expansion of the road network around the country (Fig 3) [55]. Similarly, revival of the old metre gauge rail network, construction of new commuter rail systems in Nairobi, and over 500 km of a standard gauge railway from Mombasa to Naivasha via Nairobi occurred. However, the rail system has limited use due to limited coverage and connectivity [56].

Available literature shows that in the capital city, Nairobi, walking and *matatus* (small, privately-owned buses and vans) dominate passenger transport. Eighty-three per cent of all trips include walking as a mode of travel; with 41% of the trips in the city comprising of walking only and 42% by other modes (mainly buses and *matatus*). Those trips that do not include walking (17%), are by either passenger car or motorcycle [57, 58]. Additional data collected in 2013 showed that in other major towns (Mombasa, Kisumu, Nakuru, and Eldoret), more than 37% of commuters use motorized transport while in smaller towns, majority walk [59]. The use of *matatus* is common, accounting for 88% of all motorized transport in major towns and 72% in the smaller towns. As cities increase in size, the use of *matatus* increases and the proportion of people relying on walking falls [59]. In urban slums, among working adults, over 65% walk to work, 2% use bicycles, 32% use *matatus* while 96% of school-going children walk and 4% ride a *matatu* [60]. The different modes of transport are likely to be driven by ownership of transport assets in Kenya. Nationally, only 15% of all households own a bicycle, 9% own a motorcycle, and 8% own a vehicle (car, bus, lorry, truck, three-wheeler truck, and *tuk tuk*) [61]. These vary across counties (Table 1).

**Table 1:** Percentage distribution of households by ownership of bicycles, motorcycles, cars and other means of vehicular transport (truck, lorry, bus, three-wheeler truck, and *tuk tuk*) by county [61].

ID	County Name	Bicycle	Motor Cycle (*Boda-boda*)	Car	Other vehicular	Total
	*National*	*15*	*9*.*2*	*6*.*3*	*1*.*4*	*31*.*9*
1	Mombasa	12.1	5.5	6.8	2.4	26.8
2	Kwale	19	10.6	2.3	1.2	33.1
3	Kilifi	15.3	10.4	2.8	1.4	29.9
4	Tana River	9.6	9.7	1.6	0.9	21.8
5	Lamu	28.5	15.7	1.4	0.8	46.4
6	Taita-Taveta	18.6	15.2	3.5	1.2	38.5
7	Garissa	3.5	3.2	4.1	3.7	14.5
8	Wajir	2.2	3.1	2.5	2.9	10.7
9	Mandera	3.3	6.4	3	3.5	16.2
10	Marsabit	1.9	5.6	2.2	1.2	10.9
11	Isiolo	4.2	7.9	4	1.3	17.4
12	Meru	8.7	9.8	5.2	1	24.7
13	Tharaka-Nithi	18.2	13.6	5	1	37.8
14	Embu	17.5	13.9	6.6	1.3	39.3
15	Kitui	22	13.2	3.7	1.3	40.2
16	Machakos	19.7	9.8	8.7	1.6	39.8
17	Makueni	29.9	14.2	4.5	1.3	49.9
18	Nyandarua	20.3	11.2	5.4	1.5	38.4
19	Nyeri	11.7	8.8	7.7	1.3	29.5
20	Kirinyaga	21.1	14.6	6	1.4	43.1
21	Murang’a	12.1	8.5	5	1.2	26.8
22	Kiambu	16.3	6.9	12.4	2	37.6
23	Turkana	4.2	4.8	1.3	0.8	11.1
24	West Pokot	3.2	5.8	1.6	0.7	11.3
25	Samburu	5.1	6.5	2.6	1	15.2
26	Trans Nzoia	20.6	12.7	4.9	1.3	39.5
27	Uasin Gishu	18.2	10.4	8.5	1.5	38.6
28	Elgeyo-Marakwet	3.7	6.9	3.1	1	14.7
29	Nandi	10.7	11.1	4.4	0.9	27.1
30	Baringo	7.1	8.2	4.1	0.9	20.3
31	Laikipia	21.1	13.3	6.4	1.3	42.1
32	Nakuru	19.1	10.9	7.1	1.4	38.5
33	Narok	3.7	10.7	3.5	1.1	19
34	Kajiado	11.9	9.7	10.9	1.7	34.2
35	Kericho	4.5	8.4	4.9	1	18.8
36	Bomet	5.5	12	3.7	0.8	22
37	Kakamega	24.4	11.5	3.3	1	40.2
38	Vihiga	11.5	7.8	3	0.8	23.1
39	Bungoma	26.2	11.9	3.1	1	42.2
40	Busia	34.3	11.8	2.9	0.9	49.9
41	Siaya	32.7	11.8	2.9	1	48.4
42	Kisumu	22	10.3	5.3	1.6	39.2
43	Homa Bay	12.8	10.7	2.6	1.1	27.2
44	Migori	11.5	13.2	2.9	1	28.6
45	Kisii	3.5	7.8	3.4	0.9	15.6
46	Nyamira	3.9	8.3	3.4	1	16.6
47	Nairobi	12.5	4.3	12.9	1.6	31.3

Kenya has experienced tremendous growth in the number of motorcycles popularly known as “*boda-boda*” (border-to-border) [62] that are used for private and public transport. The growth can be partly attributed to the government’s 2008 policy that zero-rated motorcycles below 250cc thereby providing an affordable alternative source of livelihood for many unemployed Kenyans especially young men [43]. It’s now a common and flexible mode of transport accessed via *boda-boda* stages or a mobile application that has made public motorized transport more accessible in the country [43].

## Methods

### Data assembly

#### Urban centres

The definition of what constitutes an urban centre varies by country and over time [10, 19, 41]. In general, urban centres are populous and densely settled areas that host high level economic and administrative functions [10]. The major country differences in the definition of urban centres involves factors like the minimum population plus a host of other measures like the presence of non-agricultural activities, population density, major type of economic activities, commercial importance, and residents’ occupation [19, 41].

In Kenya, an urban centre is defined as an area with a high density of people and human-created structures (relative to other areas around) and which has a total population of at least 2,000 people. The country’s urban areas includes cities, municipalities, town councils, and urban councils [15, 19, 61, 63]. Urban centres are expected to have infrastructural facilities such as street lighting, markets, fire stations and waste disposal [15]. Based on this local definition, a list of all urban centres in Kenya was obtained from the 2019 Kenya census [61], before their extents were digitised from the satellite images in Google Earth Pro (Version 7.3) (Fig 1B).

#### Ancillary datasets

Secondary datasets comprising of factors that influence travel time between residential areas and urban centres were assembled including the existing road network (Fig 3), land cover, elevation (captured via digital elevation models-DEMs) and travel barriers (e.g., water bodies and protected areas).

*Road network*. People mostly travel on roads to urban areas rather than on straight lines from their homes. Therefore, we assembled Kenya’s road network using data from the Ministry of Transport that used the gold standard Global Positioning System (GPS) technique to map coverage of roads in 2016. The network was then updated via OpenStreetMap and Google Map Maker as detailed elsewhere [32, 33]. The merged data vector file was cleaned by deleting duplicates and correcting digitization errors such as overshoots and undershoots at connection points or junctions and those that extended into water bodies in ArcMap version 10.5 (ESRI Inc., Redlands, CA, USA). The final road network is shown Fig 3.

*Land use-land cover*. Where no road network existed and spaces between residential areas and a road, satellite-derived information (land cover) was used to designate the underlying geographical space that people need to traverse. The landcover information was obtained from 2016 Copernicus Sentinel-2 satellite at the 20m x 20m spatial resolution containing five classes (bare areas, built-up areas, water bodies, cultivated areas and vegetation cover areas) [64]. Sentinel-2A satellite was launched in 2015, under the Copernicus programme operated by European Space Agency and the European Union to provide high-resolution satellite data which has been used for many applications such as land cover/use monitoring [65].

*Elevation*. The slope of the land impedes walking and bicycling speeds [66–69] and was obtained from Shuttle Radar Topographic Mission Digital elevation models at the 30m x 30m resolution [64]. The walking speeds were corrected according to Tobler’s formulation (Eq 1), an exponential function that describes how human walking speed varies with slope [66, 67]. Bicycling power correction assumes increased speed due to negative slope does not exceed twice the speed on flat surfaces [68, 69].

W=6*exp[‐3.5*abs(S+0.05)]whereWistheadjustedspeedandSistheslopeoftheterrainderivedfromaDEM. Eq 1

*Travel barriers*. Barriers included in the study were major rivers, lakes, forested areas, national parks and protected areas. They were considered impassable except in the presence of a bridge where a road intersected a large water body [70–72].

### Travel scenarios

Updated literature on how people travel by modes of transport from their residential areas to urban centres in Kenya is sparse. Leveraging on the healthcare spatial accessibility literature on Western Kenya, it is evident that Kenyans accessed health services either through walking, bicycling, motorised transport or a mix of walking, biking and motorised transport [73]. These transport modes are likely influenced by ownership of transport assets across Kenya’s counties (Table 1) and correlates with the discussed 2013 survey describing physical access to urban areas [57–59].

Consequently, seven travel scenarios that people in Kenya are likely to use were considered including walking only (scenario 1—the least optimistic), bicycle only (scenario 2), motorcycle only (scenario 3), vehicle only (scenario 4—most optimistic), walking followed by motorcycle transport (scenario 5), walking followed by vehicle transport (scenario 6), and walking followed by motorcycle and then vehicle transport (scenario 7). Walking is a dominant mode of transport among low-income residents and was thus combined with each mode based on previous findings [57–59], low ownership of motorized transport [61], and the fact that those beneath the poverty line are likely to walk up to 15 kilometres [74]. For a combined travel scenario (for example, scenario 7); it is assumed that from a residence, a person will first walk across areas where no road network or motorcycle passable path exists (with variable walking speeds depending on the land cover type); then take a motorcycle at the nearest motorcycle passable path or rural road to the nearest higher-class road from where the person will take a vehicle to town. However, for residential areas that are adjacent to a higher-class road, vehicular speeds automatically apply in the same way walking speeds would be applied in areas with no road network. We consider scenario 7 as the most pragmatic scenario given that majority of Kenyans are likely to use it because it combines the three common modes of walking, motorcycle, and vehicular transport [57–59, 61, 74].

### Modelling travel time

To model travel time to the nearest urban centre, the *“accessibility module”* of AccessMod software (version 5.6.3) was used [67]. AccessMod utilises the terrain-based least-cost path distance calculation to model travel time and has been widely used in healthcare applications [30, 32, 75]. The *“merge land cover module”* in AccessMod, was then used to overlay and merge the road network, landcover, rivers, lakes and protected areas to obtain a single raster dataset to which the seven travel scenarios were applied.

Speeds were assigned to each road class, landcover type and transport scenario based on a review of spatial model parameterisation from previous comparable studies in Kenya [32–35, 73, 76] (S1 Appendix). As a sensitivity analysis, each travel speed was varied by more or less than twenty percent (±20%); a cut-off routinely used [30, 77] to reflect possible variations (while travelling due to weather, traffic, car type, personal preferences, time of the day and other differentials) and to define an upper and lower bound of uncertainty [30, 77]. The analysis was conducted at 1 km spatial resolution.

### County level metrics and correlation with SDGs indicators

Since Kenya adopted a devolved government structure in 2010 (with a central government and 47 semi-autonomous county governments); counties (Fig 1A) have been used for development planning [27, 78]. Therefore, there has been an increasing need for county level metrics to aid planning, decision making, and resource allocation in Kenya. Consequently, our gridded surfaces were used to compute the mean travel time to urban centres and the corresponding uncertainty metrics by county. Kenya’s population density map, constructed using dasymetric spatial modelling techniques [79, 80] was obtained from Worldpop portal [81] and adjusted to match Kenya’s 2019 census results at sub-county level [61, 82]. The proportion of the total population within 1-hour and 2-hours of the nearest urban centre was then extracted as routinely used in spatial healthcare access analyses [30–35].

Finally, we explored how the computed metrics (proportion of the population within 1-hour of an urban centre for the pragmatic scenario) correlated with SDG indicators [83]. We considered one indicator for each of the ten SDGs for which data were available and were spatially congruent. The indicators were based on the global indicator framework by the Inter-agency and expert group on SDG indicators [84]. The ten indicators included; overall poverty, wasting, under-five mortality, literacy, access to safe and clean water, electricity connections, unemployment rate, mobile phone ownership, birth registration and internet usage. These indicators are defined in Table 2. The data used to define the indicators were obtained from the 2019 Kenya population census and household sample surveys from the different years indicated in Table 2 [61, 85, 86].

**Table 2:** Selected Sustainable Development Goals (SDGs) indicators that were correlated with spatial access metrics to the nearest urban centre in Kenya.

SDG	Indicator	Contextual definition	Year
1	1.2.1	Monthly adult equivalent total consumption expenditure per person is less than Ksh 3,252 in rural and peri-urban areas and less than Ksh 5,995 in core-urban areas	2016
	Overall Poverty		
2	2.2.2	Low weight for height. A z-score -2SD from the median of the reference population.	2016
	Wasting		
3	3.2.1	The probability that a child will die before reaching the age of five expressed per 1000 live births	2014
	Under mortality rate		
4	4.6.1	The percentage distribution of population aged 15 years and above and are capable of reading and writing.	2016
	Literacy		
6	6.1.1	The proportion of population using safely managed drinking water services	2019
	Water access		
7	7.1.1	The percentage distribution of conventional households using electricity as the main type of lighting	2019
	Electricity connectivity		
8	8.5.2	Proportion either seeking work or reported lack of work/job opportunities	2019
	Unemployment rate		
9	9.c.1	The proportion of population aged 18 years and above with a mobile phone	2016
	Mobile phone ownership		
16	16.9.1	Notified births in the last five Years	2019
	Birth registration		
17	17.8.1	Distribution of population aged three years and above using Internet	2019
	Internet usage		

The collated indicators were based on the available data at the time of analysis.

Spatial data manipulation and cartographies were done in ArcMap 10.5 (ESRI Inc., Redlands, CA, USA) while the statistical comparisons were conducted in R software (V.3·4·1).

## Results

A total of 307 urban centres were retrieved from the 2019 Kenya population census [61] and their boundaries digitised (Figs 1B and 2). Nationally, 14.8 million people or 31% of the total population resided within these urban areas. Nairobi and Mombasa counties (cities) were completely urban in 2019. With a population of 4.4 million and 1.2 million respectively; these two cities accounted for the highest share of Kenya’s urban population. In addition to Nairobi and Mombasa, other major urban centres with a population of at least a quarter a million were Nakuru, Ruiru, Eldoret, Kisumu, Kikuyu and Thika. Twenty-two (about 7%) of the 307 urban centres had a population of over 100,000 each while a third (101) had much smaller populations ranging between 2,000 and 5,000 people.

The spatial location of the urban centres largely followed the distribution of Kenya’s population (Figs 1A and 2). Majority of the urban centres were located around Lake Victoria, central and western highlands, on the Nairobi-Meru corridor, along the Mombasa-Kisumu railway/highway and along the Indian Ocean. The rest of the country had a few scattered urban centres (Figs 1A and 2). Nine counties (Nairobi, Kiambu, Mombasa, Nakuru, Kajiado, Uasin Gishu, Kisumu, Machakos and Kilifi) contained 79 (29%) urban areas and over 70% of Kenya’s urban population. Conversely, the nine arid counties (Baringo, Garissa, Isiolo, Mandera, Marsabit, Samburu, Tana River, Turkana and Wajir counties) host 49 (15%) urban centres and account for only 8% of Kenya’s urban population.

Nationally, the mean travel time to nearest urban centre was 270.4 minutes [20% uncertainty range: 225.3 to 338.1 minutes] for the least optimistic scenario (walking only) and 62.8 minutes [52.2–78.6 minutes range] for the most optimistic scenario (vehicle only) with the rest of the scenarios being between these two extremes (S2 Appendix). For the pragmatic scenario (a combination of walking followed by motorcycle and then vehicular transport), the average travel time to the nearest urban area was 87.4 minutes [72.8–109.4 minutes range]. Across the counties, the mean walking time ranged between 26.3 [21.9–33.0] minutes in Nairobi county to 795.9 [663.2–995.0] minutes in Marsabit county. Three counties had a mean walking time of less than 1-hour while 15 counties had a mean walking time of over 5 hours (S2 Appendix). Based on the most optimistic scenario, the most marginalised counties with an average travel time of more than 2-hours included Marsabit, Tana River, Turkana, Isiolo, Garissa, Wajir, Lamu, and Kitui counties. In addition to these eight counties, the pragmatic scenario, also identified Samburu, Mandera, and West Pokot counties as having an average travel time of more than 120 minutes to the nearest urban area (S2 Appendix).

**Fig 4:**
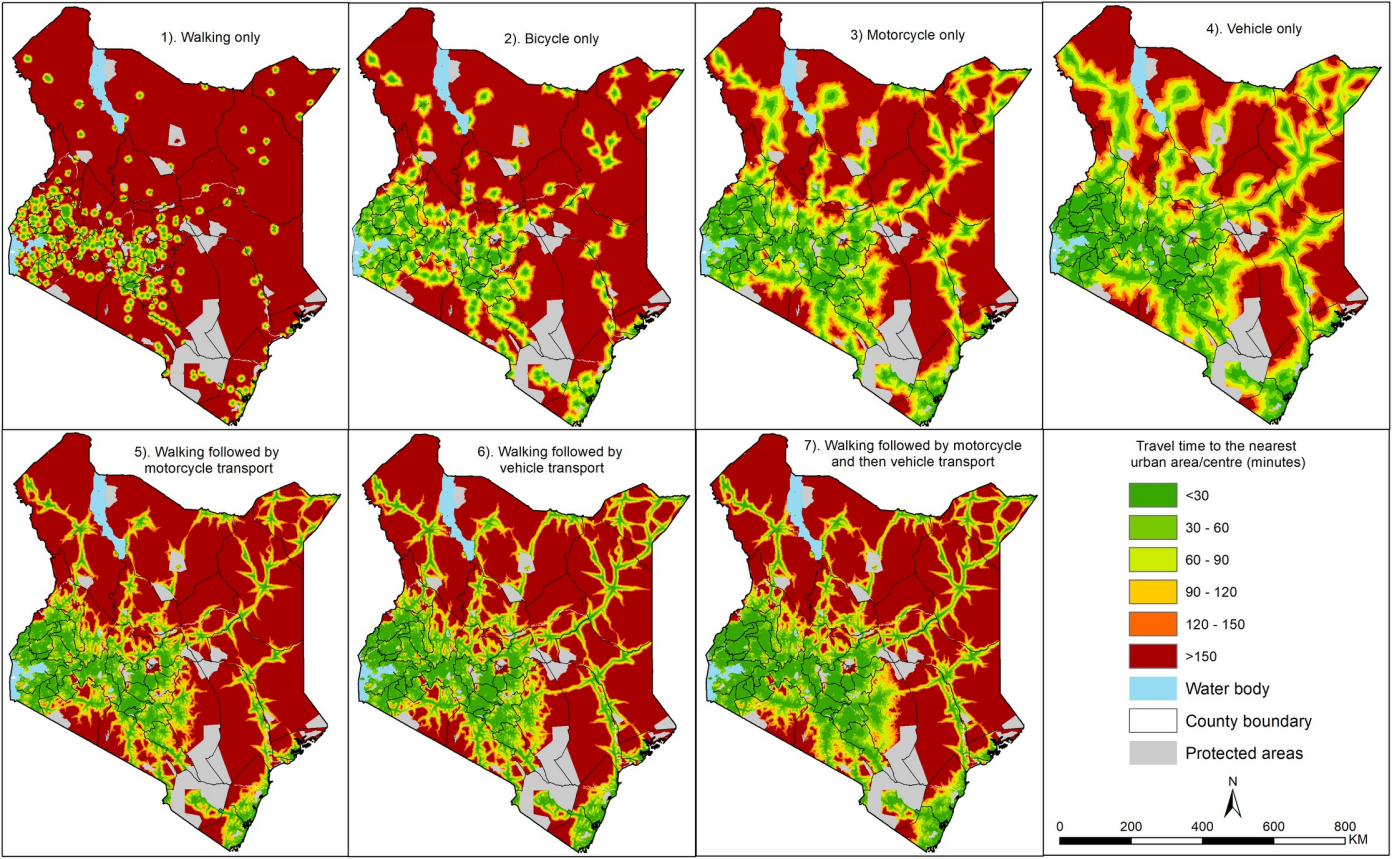
Travel time (spatial accessibility) to the nearest urban area for seven travel scenarios classified into bins of thirty minutes highlighting areas with better access (green) to the most marginalised (brown). Walking only (scenario 1), bicycle only (scenario 2), motorcycle only (scenario 3), vehicle only (scenario 4), walking followed by motorcycle transport (scenario 5), walking followed by vehicle transport (scenario 6), and walking followed by motorcycle and then vehicle transport (scenario 7). Source: authors.

Over twenty-one million people or 44.9% of Kenya’s 2019 population resided within 1-hour travel time of an urban centre for walking scenario. The range of the population within 1-hour of an urban area was 39.8% to 50.0%. Conversely, 41.6 million people or 87.4% [range 84.4–89.2] of the total population resided within 1-hour of travel time of an urban centre for the most pragmatic scenario. Geographically marginalised areas under the least optimistic and most pragmatic scenarios, comprised of 16.0 million people (33.6%) and 3.2 million people (6.7%) who had travel times of more than 2-hours to the nearest urban area respectively. When considering the most optimistic scenario, 43.4 million were within 1-hour of the nearest urban area while 2.2 million were outside the 2-hour threshold. Fig 4 shows maps of spatial access to the nearest urban centre based on all seven travel scenarios binned into classes of 30 minutes; with the most marginalised areas shown in red and brown colours.

Spatial access to the nearest urban centre across each of the 47 counties was highly heterogeneous. The proportion of the population within 1-hour of an urban centre ranged between 9.7% [8.0%-11.8%] in Kitui county to 99.6% [99.2%-99.8%] in Nairobi county for the least optimistic scenario and between 24.2% [21.5%-27.2%] in Wajir county and 100% in seven counties for the most pragmatic scenario. A similar range was observed for the most optimistic scenario, ranging between 31.6% [26.6%-36.8%] in Wajir county to 100% in several counties (Table 2). The most marginalised counties with more than 50% of people living outside of a 2-hour travel time from an urban centre for the least optimistic scenario included Wajir, Kitui, West Pokot, Turkana, Samburu, Tana River, Narok, Elgeyo-Marakwet, Baringo, Mandera, Garissa, Marsabit, and Makueni. However, when considering the most pragmatic scenario, only Garissa, Turkana and Wajir had over 50% of its population outside this threshold. An additional eight counties including Marsabit, Tana River, Mandera, West Pokot, Samburu, Isiolo, Lamu, Narok, Baringo had at least 10% of the population outside the 2-hour travel time. The proportion of the population within 1-hour of an urban centre for each travel scenario is shown in Table 3, while those within 2-hours are shown in S3 Appendix.

**Table 3:** The proportion of proportion within 1-hour travel time of the nearest urban centre in Kenya in 2019 for seven travel scenarios; walking only (scenario 1), bicycle only (scenario 2), motorcycle only (scenario 3), vehicle only (scenario 4) walking followed by motorcycle transport (scenario 5), walking followed by vehicle transport (scenario 6) and walking followed by motorcycle and then vehicle transport (scenario 7).

Province	ID	County Name	Scenario 1	Scenario 2	Scenario 3	Scenario 4	Scenario 5	Scenario 6	Scenario 7
Coast	1	Mombasa	98.56[98.23–98.96]	99.51[99.45–99.55]	93.82[93.65–94.13]	94.13[93.81–94.13]	99.51[99.24–99.52]	99.5[99.32–99.55]	99.54[99.53–99.55]
	2	Kwale	29.82[25.29–34.33]	60.79[50.48–68.45]	84.48[72.55–91.44]	94.18[88.15–96.65]	75.68[65.11–83.49]	82.98[74.24–88.52]	92.25[85.53–95.15]
	3	Kilifi	40.10[35.5–45.19]	69.34[61.76–75.59]	81.95[76.29–86.14]	88.23[83.78–91.34]	82.51[76.11–87.16]	87.64[82.03–91.21]	90.43[86.05–93.55]
	4	Tana River	15.41[12.06–17.68]	27.53[23.41–32.16]	41.32[34.44–47.82]	55.02[47.65–61.64]	34.09[28.01–40.13]	40.11[34.4–45.16]	40.38[34.69–45.53]
	5	Lamu	43.12[39.09–49.39]	59.19[56.23–61.96]	64.75[60.37–67.19]	68.54[65.51–70.78]	57.59[52.9–62.66]	58.02[53.98–63.18]	62.97[59.69–66.95]
	6	Taita Taveta	38.37[33.48–43.75]	74.84[64.26–81.26]	91.8[83.51–96.31]	97.93[94.63–99.17]	88.25[82.15–91.36]	91.27[87.97–94.03]	93.62[90.59–95.78]
North Eastern	7	Garissa	27.95[25.44–29.7]	33.18[31.26–34.85]	36.61[34.33–38.93]	40.91[38.28–43.32]	33.06[28.54–35.18]	34.55[29.93–36.72]	35.35[30.32–37.52]
	8	Wajir	15.12[14.21–15.92]	19.13[17.56–20.75]	24.08[21.23–27.05]	31.55[26.63–36.82]	21.32[19.37–23.33]	24.05[21.43–27.02]	24.21[21.47–27.23]
	9	Mandera	23.96[22.52–26.37]	32.37[30.01–34.78]	40.23[35.5–45.67]	52.72[44.49–60.44]	36.33[31.97–40.28]	40.74[35.72–45.67]	40.72[35.72–45.67]
Eastern	10	Marsabit	27.69[26.49–28.95]	35.31[32.61–37.97]	42.56[38.52–46.7]	50.62[45.7–54.45]	37.94[34.82–40.96]	41.61[37.98–44.6]	41.61[37.98–44.6]
	11	Isiolo	43.95[42.76–45.88]	51.95[49.06–54.48]	53.95[50.49–57.16]	60.46[55.79–65.19]	54.85[51.84–57.97]	57.58[54.63–60.8]	57.77[54.73–61.09]
	12	Meru	35.51[26.95–42.81]	75.53[66.59–82.54]	94.49[84.9–97.54]	98.5[95.71–99.31]	89.9[83.16–93.66]	92.78[87.76–95.31]	97.35[95.34–98.26]
	13	Tharaka-Nithi	27.05[19.3–35.23]	62.55[53.61–69.52]	81.49[72.28–90.09]	92.84[83.78–99.99]	75.26[67.53–80.93]	80.49[73.59–86.43]	91.19[82.78–95.29]
	14	Embu	27.47[21.4–34.1]	66.44[54.78–74.83]	85.9[78.55–90.59]	93.3[87.95–96.67]	82.31[75.65–87.49]	88.05[82.93–92.23]	93.74[88.94–97.06]
	15	Kitui	9.71[7.96–11.84]	26.03[19.96–31.59]	42.9[34.26–49.83]	57.73[49.1–66.6]	40.54[32.65–46.99]	50.21[42.43–57.79]	57.65[49.1–66.36]
	16	Machakos	41.76[34.74–48.23]	76.21[67.46–82.4]	89.45[82.7–93.31]	95.95[92.02–98.45]	84.74[78.94–88.66]	89.8[84.8–93.4]	96.41[92.64–98.25]
	17	Makueni	12.32[9.29–16.59]	46.22[33.39–57.49]	73.88[59.75–82.26]	84.98[77.13–90.84]	65.95[53.83–74.38]	73.4[63.65–79.38]	86.35[79.42–92.08]
Central	18	Nyandarua	36.56[29.56–46.98]	83.14[72.09–90.3]	98.63[91.8–99.81]	99.88[99.27–99.93]	95.77[89.54–97.63]	95.69[91.02–97.54]	98.75[98.05–99.22]
	19	Nyeri	55.89[45.14–66.33]	92.58[85.13–95.85]	99.29[96.82–100.17]	100[99.88–100]	97.35[94.25–98.89]	98.51[97.03–99.28]	99.44[99.22–99.53]
	20	Kirinyaga	67.43[54.29–79.88]	99.43[95.48–99.81]	99.92[99.79–99.93]	99.93[99.92–99.93]	98.64[97.29–99.51]	99.34[98.02–99.9]	100[100–100]
	21	Murang’a	31.9[24.44–40.48]	81.94[66.33–92.35]	98.11[94.69–99.78]	100[98.84–100]	97.68[96.16–98.65]	98.69[97.65–99.12]	99.07[98.03–99.77]
	22	Kiambu	77.13[69.02–84.98]	96.78[93.81–98.89]	99.74[98.21–100.21]	100[99.91–100]	99.57[98.9–99.8]	99.72[99.32–99.86]	99.93[99.84–99.92]
Rift Valley	23	Turkana	18.28[17.41–19.06]	22.23[20.59–23.71]	28.12[25.05–31.49]	36.82[31.45–41.35]	23.76[21.49–26.27]	26.79[24.03–29.76]	26.8[24.03–29.76]
	24	West Pokot	11.72[9.92–14.25]	26.91[21.96–31.49]	40.09[32.35–48.41]	57.83[46.34–67.7]	35.76[29.52–41.83]	44.04[36.95–50.23]	46.01[38.91–52.52]
	25	Samburu	10.84[8.44–13.19]	24.25[18.15–30.14]	38[29.4–47.18]	53.96[42.33–64.68]	32.3[25.93–39.48]	39.67[31.42–47.73]	39.67[31.42–47.73]
	26	Trans Nzoia	26.62[21.8–30.48]	65.35[51.08–76.56]	92.13[81.25–98.38]	99.72[94.09–100]	86.66[76.67–92.73]	92.46[85.17–96.34]	97.76[93.48–98.94]
	27	Uasin Gishu	55.1[50.37–59.46]	82.83[74.35–89.3]	95.3[89.22–97.96]	98.6[96.33–98.8]	93.14[86.62–96.73]	95.97[91.61–98.22]	99.41[98.69–99.59]
	28	Elgeyo-Marakwet	12.94[10.13–16.16]	42.74[31.13–54.46]	65.87[49.38–79.56]	85.7[69.57–93.85]	61.63[46.64–75.11]	74.89[59.56–83]	82.37[69.24–88.65]
	29	Nandi	23.08[17.11–29.64]	60.96[47.36–74.53]	91.88[75.67–97.22]	98.63[94.06–99.43]	87.11[74.08–93.74]	91.9[84.89–95.85]	96.37[92.71–98.22]
	30	Baringo	17.2[14.77–19.49]	33.77[28.06–39.92]	54.37[44.33–62.38]	68.16[57.51–76.52]	50.67[40.99–58.31]	60.19[50.65–66.12]	65.37[56.21–71.17]
	31	Laikipia	41.65[38.49–44.38]	64.35[55.76–73.02]	87.38[76.67–92.03]	93.92[89.8–96.09]	77.57[68.93–82.37]	81.22[75–85.86]	88.94[85.36–91.33]
	32	Nakuru	57.31[50.52–62.88]	81.42[74.17–86.31]	93.96[89.29–96.53]	97.79[94.64–99.52]	95.02[91.43–96.53]	97.01[94.84–98.5]	97.2[95.1–98.78]
	33	Narok	14.03[11.95–16.46]	31.05[24.13–37.7]	47.01[36.58–57.16]	62.58[49.66–73.08]	42.87[34.49–49.8]	48.69[41.02–55.96]	58[47.1–67.3]
	34	Kajiado	43.46[40.85–46.19]	58.44[53.85–62.81]	69.33[63.44–74.53]	79.02[72.1–84.72]	61.28[55.96–66.28]	66.19[59.89–71.18]	72.9[66.84–78.07]
	35	Kericho	32.53[25.71–40.06]	80.44[65.89–90.93]	93.7[88–96.8]	97.88[94.61–98.57]	93.02[88.6–96.21]	95.29[91.87–97.68]	98.05[96.08–98.93]
	36	Bomet	20.41[14.99–25.79]	63.17[46.15–78.01]	87.89[75.39–94.74]	98.13[90.78–99.78]	89.17[80.01–95.52]	96.17[88.53–98.28]	98.72[95.45–99.17]
Western	37	Kakamega	40.14[32.1–49.11]	88.58[74.96–96.48]	100[98.19–100]	100[100–100]	96.84[93.29–98.49]	97.53[94.31–98.66]	99.26[99–99.31]
	38	Vihiga	67.3[54.61–80.12]	99.52[98.24–99.7]	100[100–100]	100[100–100]	99.62[99.47–99.85]	99.74[99.47–99.93]	99.74[99.62–99.94]
	39	Bungoma	37.74[29.98–45.38]	88.43[74.81–93.87]	99.78[95.99–100]	100[99.95–100]	98.71[95.2–99.51]	98.89[97.28–99.55]	99.43[99.07–99.65]
	40	Busia	33.73[27.5–40.42]	86.35[69.43–94.98]	100[96.57–100]	100[100–100]	96.14[92.13–98.11]	97.45[94.88–98.41]	97.61[96–98.63]
Nyanza	41	Siaya	30.87[23.65–38.71]	75.7[63.54–84.41]	90.8[85.7–93.66]	94.42[91.51–95.65]	90.68[86.09–93.9]	94.83[90.07–95.53]	95.31[91.31–95.55]
	42	Kisumu	64.25[57.85–69.32]	91.58[84.87–95.96]	98.1[96.1–98.87]	98.94[98.43–98.94]	98.3[95.77–99.09]	98.84[97.51–99.42]	99.52[98.75–99.73]
	43	Homa Bay	29.81[23.98–34.92]	73.52[57.18–85.29]	96.37[87.5–98.84]	99.08[97.45–99.37]	88.8[82.14–91.89]	93.95[87.01–93.35]	95.41[89.38–94.09]
	44	Migori	36.93[30.01–43.76]	78.23[67.4–85.82]	93.09[86.28–97.6]	99.11[94.23–100]	89.86[82.87–94.68]	94.39[88.03–96.86]	96.79[92.45–98.48]
	45	Kisii	57.65[48.81–65.83]	98.11[88.41–100]	100[100–100]	100[100–100]	99.86[99.21–100]	99.91[99.6–100]	99.99[100–100]
	46	Nyamira	36.87[27.48–45.49]	99.22[87.27–99.96]	97.53[97.49–97.56]	97.56[97.53–97.56]	99.93[99.66–99.98]	99.91[99.62–99.98]	100.01[99.98–99.98]
Nairobi	47	Nairobi	99.56[99.17–99.77]	99.93[99.91–99.93]	99.65[99.64–99.65]	99.5[99.4–99.5]	99.91[99.88–99.92]	99.91[99.89–99.93]	99.92[99.93–99.99]
National	48	National	44.87[39.73–49.98]	72.58[64.71–78.07]	84.09[78.82–87.23]	89.07[85.6–91.27]	81.72[77.05–84.79]	84.78[80.87–87.17]	87.36[84.39–89.24]

The mean speed was varied by ±20% to define an upper and lower bound of uncertainty.

Across the former administrative provinces of Kenya, North-eastern was the most marginalised. More than two-thirds of the population in this region still lives outside of 1-hour travel time of an urban centre compared to all the other regions which had less than a quarter of their population outside the 1-h threshold based on the most pragmatic scenario (Table 3). However, when the time threshold criterion was relaxed, half of the population was outside the 2-hour zone in North-eastern and less than 15% in the rest of the regions (S3 Appendix).

The correlation coefficients between the proportion of the population within 1-hour travel time of an urban centre for the most pragmatic scenario and each of the ten SDG indicators exhibited moderate to strong [83] positive or negative correlations which were all significant at p <0.05 except for the under-five mortality (p = 0.0834) (S4 Appendix).

## Discussion

We have presented updated and localised estimates of spatial accessibility scores to urban centres in Kenya for 2019 at a high spatial resolution for seven travel scenarios. The gridded surfaces capture the local definition of an urban area and local modes of transport especially walking, bicycling and motorcycling. The results are therefore, more suitable for local policy-making; unlike the global surfaces which are generalized and generally inapplicable to local contexts [2, 4, 39, 40]. The recent global surfaces presented a single scenario combining several modes of transport ignoring the individual modes of transport considered in this analysis. Further, a third (101) of the urban centres in Kenya would have been missed or unaccounted for, if the arbitrary urban population cut-off of 5,000 people would have been used in the Kenyan context.

The most pragmatic travel scenario combining walking followed by motorcycle and vehicular transport, shows that approximately one in every 15 Kenyans (3.2 million people or 6.7% of the total 2019 population) was marginalised or residing more than 2-hours of travel time from the nearest urban centre. Assuming a walking only scenario presents a dire situation where one in three individuals nationally lives beyond the 2-hour threshold and is therefore deprived of the resources, services and opportunities that they need which are usually available in urban areas. If the entire population had access to a bicycle, those deprived of resources would reduce from 33% (for walking) to 12%. Universal access to motorcycles and vehicles would further reduce the deprived to 7% and 4.6% respectively. These access scores have practical implications on access to services in Kenya.

Within Kenya, 93% of the over 72,000 educational facilities mapped in 2007 [5] are within one 1-hour travel time of the nearest urban centre for the most pragmatic scenario. Likewise, 85% of the over 6,000 public health facilities assembled in 2019 [6] and 99% of over 60,000 financial institutions (such as commercial bank branches, automated teller machines (ATMs), Savings and Credit Co-Operative Societies (SACCOS), post banks, Foreign Exchange Bureaus (forex bureaus) and mobile money agents mapped in 2013 are located within the same time thresholds [7]. This implies that over 6 million people (12.6% of country’s population) living beyond the 1-hour travel time for the most pragmatic scenario (Table 3) are served only by 7% of the educational institutions, 15% of the health care facilities and 1% of the country’s financial institutions. The proliferation of mobile money platforms especially *MPESA* agents which are widely distributed in the country’s rural and urban areas has substantially reduced the need to access a physical bank. However, there are still some services that require access to physical financial institution usually located in urban areas.

National urban access averages mask widespread heterogeneities across Kenya’s counties. When considering the most pragmatic scenario, thirty-six counties out of 47 had over 90% of their population within a 2-hour travel time to an urban area. A further six counties had at least two-thirds of their population within this threshold. The most marginalised counties with at least 37% of their population outside a 2-hour travel time of an urban area included Wajir, Turkana, Garissa, Marsabit, Tana River and Mandera counties. Combined, these six counties, accounted for over two-thirds of the marginalised population in this study. Despite Kitui, Kajiado, Baringo, Narok, Isiolo, Samburu, and West Pokot having lower proportions outside 2-hours of travel time to an urban area; the corresponding magnitude of the population ranged between 76,595 in Isiolo to 198,307 people in West Pokot county. These counties are characterized by a sparse population, have a lower number of urban centres relative to their large geographical areas, demonstrated lack of access to a motorable road network, and have a much lower ownership of bicycles, motorcycles and vehicles.

Eleven counties (Turkana, Mandera, Isiolo, Marsabit, Tana River, Garissa, Wajir, West Pokot, Kilifi, Lamu and Samburu) were previously identified as marginalised areas through individual and group questionnaires by the CRA [28]. These counties have large populations outside the 2-hour threshold (S3 Appendix). This not only provides a validation mechanism but reiterates the need to prioritize these counties during resource allocation and planning so that they can improve their road networks. Initiatives such as mobile clinics and markets can also be used as points of entry to bring services closer to the population in these counties. For example, most of the government service hubs (*huduma centres*) launched in 2013 to provide access to common government services are located in major urban centres [87, 88]. Hence those in the rural areas have to travel for long distance/periods to access services such as renewal of driving licences, student loan services, issuance of police abstracts, and business permit [87, 88]. While some of these services are available in the E-Citizen government portal, this requires access to an internet connection which is often lacking in the marginalized areas. Additionally, certain functions such as document verifications, finger printing and document collection require physical access to government centers. Appreciating this need, the government introduced *Huduma Mashinani* (service at the grassroots) in 2017, a program for providing outreach services once a month in each sub-County and specific outreaches to specialized groups of people in prisons, schools, hospitals and children’s homes [87–89].

The marginalised counties of northern Kenya have historically been associated with poor development indicators including limited geographic access and utilisation of health care services, low public health intervention coverage and increased social vulnerability [30, 32, 90–96]. The region has long been less served with schools, safe water, health facilities and paved roads [28, 97] which are key in defining marginalization [28]. In such areas, an improved road network will improve access to public services besides bringing services closer to the people.

The significant, high to moderate correlation coefficients between the SDG indicators and spatial access to urban areas provides further evidence that being physically far from an urban area is correlated with higher rates of poverty, wasting, low literacy, low electricity connectivity, high unemployment rates, low mobile phone ownership, poor birth registration and low internet usage. Therefore, marginalized and remote areas suffer more SDG negative outcomes than urban areas and adjacent neighbourhoods. Even though these results are exploratory and do not infer causality between poor spatial access and poor coverage of some of the SDG indicators, the accessibility metrics are a proxy for remoteness and spatial marginalization that has important implications across several SDG themes.

For health and wellbeing, our metrics can aid in planning for public services and resources [21, 22] such as the density of nurses [22], response to disease outbreaks and pandemics [98] and for characterizing vulnerability [20]. In the energy sector, spatial access metrics have been used in the decentralized rural electrification programme in Kenya to speed up universal power access [53]. Spatial access to markets in urban areas facilitates income security [13, 14] while areas that are marginalized from urban areas have higher income insecurity. Spatial access to markets is vital in income security given that most rural areas rely on agriculture for their livelihoods and generate income from sale of agricultural goods [99]. In Kenya, both the expansive road rehabilitation and consequent improved spatial access to market areas, therefore, resulted in increased agricultural production due to better market access as well as the use of inorganic fertilisers, and adoption of high-value crops between 2004 to 2012 [99].

The use of the seven travelling scenarios explored in this paper highlights different alternatives for accessing an urban centre and provides insights into the importance of Kenya’s various transport modes [8]. This is crucial because it shows how Kenya’s spatial access might be improved when road network connectivity is enhanced, households are empowered financially to afford public transport, or to increase their ownership of motorcycles and bicycles [8].

## Limitations

The metrics discussed herein have some caveats. Travel time was computed to the nearest urban centre whereas there is a good likelihood of bypassing the nearest urban centre in situations where services are not available or are deemed to be inferior. Several conditions that affect travel speeds such as weather conditions, traffic congestion, and delays [100, 101] were not accounted for due to data limitations. However, a range of speeds and their uncertainty was considered to reflect various travel occurrences. Several regions in Kenya are affected by insecurity and conflict which impede access to certain urban areas more so in North-eastern Kenya [102]. These were not accounted for in this analysis. Our study only considered physical access even though good physical access does not guarantee affordability or economic and social access to urban services [4, 103]. Our study did not discriminate between spatial access to different sized urban areas which correspond to access to different resources, services and opportunities. We did not consider rail transport which is used by a small proportion of Kenya’s population especially within Nairobi city and its environs and has limited coverage. Moreover, we did not fully interrogate the impact of mobile services e.g., mobile banking that may lessen the need to travel to urban areas for various financial services. Finally, despite the opportunities available in urban areas, access to them is socioeconomically restricted. Moreover, urbanization is not always economically productive if urban areas lack basics like good roads [17, 36–38].

## Conclusions

We have improved and updated previous urban access metrics in Kenya by localising the definition of an urban centre and accounting for local travel modes for seven travel scenarios. These access metrics are more suitable for local policy-making. Importantly the approach can be replicated in other countries or sub-regions with ease and used to generate estimates of alternative local travel scenarios combined with policy relevant thresholds for decision making.

In Kenya, the proportion of people within 1-hour of the nearest urban centre ranges between 21.3 million (44.9%) considering walking only and 42.4 million (89.1%) considering vehicle transport only while the most pragmatic scenario (a combination of walking, motorcycle and vehicle use) puts 41.6 million people (87.4%) within this threshold. This provides a basis for national government policies related to infrastructure development and public service provision and has implications for achieving several SDGs such as access to education and healthcare. Substantial variations in travel time to the nearest urban centre continues to persist within Kenya. Most of the counties have a majority of the people within 1-hour travel time of an urban area. However, counties in Northern and North-eastern Kenya are most marginalised from an urban access perspective. This is partly driven by their sparse population, poor infrastructure, and limited transport options. To enhance equitable access to services in the country, the marginalised areas should be prioritized when it comes to road development and related infrastructure provision.

## Supporting information

S1 Appendix Travel speeds used in the modelling travel time to urban centres in Kenya for seven different travel scenarios. (DOCX)

S2 Appendix Mean travel time to the nearest urban centre in Kenya in 2019 for seven travel scenarios at county level. (DOCX)

S3 Appendix The proportion of population within 2-hour travel time of the nearest urban centre in Kenya in 2019 for five seven scenarios. (DOCX)

S4 Appendix Correlation matrix between travel time to the nearest urban centre for the most pragmatic scenario (7) and ten SDG indicators. (DOCX)
