# Supplementary material for: Modelling geographical accessibility to urban centres in Kenya in 2019
Source: PLoS One. 2021 May 14;16(5):e0251624. doi: 10.1371/journal.pone.0251624 (PMC8127925; doi:10.1371/journal.pone.0251624)
Supplement: S1 Appendix — (DOCX) [file pone.0251624.s001.docx]

**S1 Appendix**

Travel speeds used in the modelling travel time to urban centres in Kenya for different travel scenarios. Walking only (scenario 1), bicycling only (scenario 2), motorcycle only (scenario 3), vehicle only (scenario 4), walking followed by motorcycle transport (scenario 5), walking followed by vehicle transport (scenario 6), and walking followed by motorcycle and then a vehicle transport (scenario 7). The speeds are based on previous comparable studies in Kenya and were varied by ±20% to define an upper and lower bound of uncertainty [1–7].

| **Road and land cover type** | **Scenario -Speed in Km/hr** | | | | | | |
| --- | --- | --- | --- | --- | --- | --- | --- |
| **Road or land cover type** | **1** | **2** | **3** | **4** | **5** | **6** | **7** |
| International and national trunk roads | 5 | 15 | 35 | 65 | 35 | 65 | 65 |
| Primary and secondary roads | 5 | 15 | 30 | 40 | 30 | 40 | 40 |
| Minor and government roads | 5 | 10 | 20 | 25 | 20 | 25 | 25 |
| Settlement Roads | 5 | 10 | 20 | 25 | 20 | 5 | 20 |
| Rural access, farm and unclassified roads | 5 | 10 | 20 | 25 | 5 | 5 | 20 |
| Trees Cover Areas | 2.50 | 5 | 6.25 | 7.5 | 2.50 | 2.50 | 2.50 |
| Shrubs cover and sparse vegetation | 5 | 7.5 | 10 | 15 | 5 | 5 | 5 |
| Grassland | 3.5 | 6.25 | 7.5 | 10 | 3.5 | 3.50 | 3.5 |
| Cropland | 3.25 | 6.25 | 7.5 | 10 | 3.25 | 3.25 | 3.25 |
| Bare areas and built-up areas | 5 | 10 | 20 | 25 | 5 | 5 | 5 |
| Open Water and regularly flooded | 0 | 0 | 0 | 0 | 0 | 0 | 0 |

**References**

1 Dixit A, Lee M-C, Goettsch B, *et al.* Discovering the cost of care: consumer, provider, and retailer surveys shed light on the determinants of malaria health-seeking behaviours. *Malar J* 2016;**15**:179. doi:10.1186/s12936-016-1232-7

2 Joseph NK, Macharia PM, Ouma PO, *et al.* Spatial access inequities and childhood immunisation uptake in Kenya. *BMC Public Health* 2020;**20**:1407.

3 Macharia PM, Odera PA, Snow RW, *et al.* Spatial models for the rational allocation of routinely distributed bed nets to public health facilities in Western Kenya. *Malar J* 2017;**16**:1–11. doi:10.1186/s12936-017-2009-3

4 Ouko JJO, Gachari MK, Sichangi AW, *et al.* Geographic information system-based evaluation of spatial accessibility to maternal health facilities in Siaya County, Kenya. *Geogr Res* 2019;**57**:286–98. doi:10.1111/1745-5871.12339

5 Ocholla IA, Agutu NO, Ouma PO, *et al.* Geographical accessibility in assessing bypassing behaviour for inpatient neonatal care, Bungoma County-Kenya. *BMC Pregnancy Childbirth* 2020;**20**:287. doi:10.1186/s12884-020-02977-x

6 Ouma PO, Agutu NO, Snow RW, *et al.* Univariate and multivariate spatial models of health facility utilisation for childhood fevers in an area on the coast of Kenya. *Int J Health Geogr* 2017;**16**:34. doi:10.1186/s12942-017-0107-7

7 Ouma PO, Maina J, Thuranira PN, *et al.* Access to emergency hospital care provided by the public sector in sub-Saharan Africa in 2015: a geocoded inventory and spatial analysis. *Lancet Glob Heal* 2018;**6**:e342–50.
