# Supplementary material for: Modelling geographical accessibility to urban centres in Kenya in 2019
Source: PLoS One. 2021 May 14;16(5):e0251624. doi: 10.1371/journal.pone.0251624 (PMC8127925; doi:10.1371/journal.pone.0251624)
Supplement: S2 Appendix — (DOCX) [file pone.0251624.s002.docx]

**S2 Appendix**

| **Province** | **County name** | **ID** | **Scenario 1** | **Scenario 2** | **Scenario 3** | **Scenario 4** | **Scenario 5** | **Scenario 6** | **Scenario 7** |
| --- | --- | --- | --- | --- | --- | --- | --- | --- | --- |
| **Coast** | Mombasa | 1 | 30.3[25.2-38.0] | 16.1[13.3-19.4] | 10.4[8.6-13.2] | 8.0[6.6-10.1] | 17.4[11.7-21.8] | 12.9[10.8-20.4] | 7.7[6.8-10.3] |
|  | Kwale | 2 | 280.9[234.0-351.3] | 136.3[113.5-170.4] | 80.5[67-100.8] | 60.5[50.3-75.8] | 148.1[123.0-185.3] | 135.6[112.9-170.3] | 73.3[61.0-91.8] |
|  | Kilifi | 3 | 396.9[330.7-496.3] | 201.8[168.1-252.4] | 123.1[102.5-154] | 91.0[75.7-113.8] | 190.0[158.2-237.6] | 170.4[141.9-213.2] | 120.7[100.5-151.0] |
|  | Tana River | 4 | 724.2[603.4-905.3] | 401.7[334.7-502.1] | 264.5[220.3-330.7] | 186.3[155.2-233.0] | 358.8[298.7-448.6] | 321.6[267.9-402.4] | 308.3[256.9-385.5] |
|  | Lamu | 5 | 538.6[448.8-673.4] | 256.0[213.3-320.0] | 187.3[156-234.3] | 140.2[116.8-175.4] | 222.1[184.6-277.7] | 197.6[164.6-247.7] | 191.6[159.7-239.7] |
|  | Taita Taveta | 6 | 272.8[227.2-341.1] | 129.1[107.5-161.5] | 112.7[93.8-141.0] | 81.0[67.4-101.4] | 119.6[99.5-149.7] | 107.7[89.7-135.0] | 82.2[68.4-102.9] |
| **North Eastern** | Garissa | 7 | 719.0[599.1-898.9] | 370.8[308.9-463.6] | 231.4[192.8-289.4] | 171.2[142.6-214.2] | 368.6[307.1-460.8] | 339.3[282.7-424.3] | 270.8[225.6-338.6] |
|  | Wajir | 8 | 676.7[563.9-846.1] | 341.3[284.3-426.7] | 210.4[175.3-263.2] | 151.2[125.9-189.1] | 292.7[243.7-366.0] | 259.2[215.9-324.3] | 258.0[214.9-322.6] |
|  | Mandera | 9 | 435.4[362.8-544.4] | 214.7[178.9-268.5] | 133.0[110.7-166.3] | 93.8[78.1-117.3] | 185.6[154.6-232.2] | 163.8[136.4-204.9] | 163.8[136.4-204.9] |
| **Eastern** | Marsabit | 10 | 795.9[663.2-995.0] | 409.3[340.9-511.7] | 246.8[205.6-308.7] | 180.9[150.6-226.2] | 384.3[320.1-480.5] | 343.8[286.4-429.9] | 343.8[286.4-429.8] |
|  | Isiolo | 11 | 541.3[451.0-676.7] | 308.0[256.6-385.1] | 197.4[164.4-246.9] | 144.4[120.2-180.6] | 306.1[254.7-382.7] | 282.3[235.2-353.5] | 282.0[235.1-352.9] |
|  | Meru | 12 | 175.4[146.0-219.3] | 74.5[62.0-93.3] | 62.7[52.2-78.5] | 46.5[38.6-58.2] | 66.7[55.5-83.5] | 58.0[48.2-72.6] | 49.2[40.9-61.6] |
|  | Tharaka-Nithi | 13 | 197.2[164.3-246.7] | 92.1[76.6-115.2] | 65.8[54.7-82.3] | 50.5[42.0-63.2] | 89.6[74.6-112.1] | 80.8[67.2-101.1] | 65.3[54.3-81.7] |
|  | Embu | 14 | 207.0[172.4-258.9] | 92.5[77.0-115.8] | 60.3[50.2-75.5] | 45.2[37.5-56.6] | 70.4[58.6-88.2] | 59.0[49.1-73.9] | 48.5[40.3-60.7] |
|  | Kitui | 15 | 640.2[533.4-800.4] | 272.6[227.0-341.0] | 202.0[168.3-252.6] | 139.6[116.3-174.6] | 198.0[164.8-247.6] | 162.3[135.1-203.1] | 132.8[110.6-166.2] |
|  | Machakos | 16 | 156.7[130.5-196.0] | 68.1[56.7-85.3] | 43.9[36.5-55.0] | 31.5[26.1-39.5] | 50.5[41.9-63.3] | 41.0[34.1-51.5] | 29.7[24.7-37.3] |
|  | Makueni | 17 | 183.0[152.4-228.9] | 86.8[72.3-108.7] | 60.4[50.2-75.6] | 44.1[36.7-55.2] | 81.6[67.9-102.1] | 72.0[59.9-90.1] | 47.7[39.6-59.7] |
| **Central** | Nyandarua | 18 | 104.2[86.8-130.4] | 45.9[38.2-57.0] | 34.1[28.4-42.8] | 25.7[21.3-32.2] | 38.1[30.1-47.8] | 33.0[27.4-43.7] | 24.3[20.3-30.8] |
|  | Nyeri | 19 | 115.4[96.1-144.4] | 53.3[44.4-66.6] | 44.7[37.1-55.9] | 34.0[28.2-42.6] | 43.2[35.9-54.1] | 37.4[31.1-46.8] | 33.1[27.5-41.5] |
|  | Kirinyaga | 20 | 97.7[81.3-122.2] | 46.7[38.9-58.4] | 38.3[31.9-48.1] | 29.8[24.7-37.3] | 52.8[43.9-66.1] | 48.2[40.1-60.4] | 40.2[33.4-50.4] |
|  | Murang'a | 21 | 102.9[85.7-128.8] | 44.8[37.3-56.1] | 33.4[27.8-41.9] | 25.1[20.9-31.5] | 32.7[27.2-41.0] | 27.5[22.8-34.5] | 24.9[20.6-31.2] |
|  | Kiambu | 22 | 79.2[65.9-99.1] | 34.4[28.6-43.1] | 24.8[20.6-31.1] | 18.4[15.3-23.1] | 24.9[20.6-31.2] | 20.8[17.2-26.1] | 16.8[13.9-21.1] |
| **Rift Valley** | Turkana | 23 | 686.7[572.1-858.5] | 339.8[283.1-424.8] | 267.5[222.9-309.4] | 192.6[160.4-240.9] | 325.3[270.4-406.7] | 289.7[241.4-363.2] | 289.1[241.0-361.8] |
|  | West Pokot | 24 | 544.8[453.9-681.2] | 254.7[212.3-318.4] | 156.3[130.2-195.5] | 112.0[93.2-140.1] | 199.2[165.8-249.1] | 168.9[140.7-211.3] | 161.4[134.4-201.9] |
|  | Samburu | 25 | 445.7[371.3-557.2] | 221.1[184.1-276.5] | 138.0[114.9-172.6] | 99.2[82.6-124.2] | 194.6[162.1-243.4] | 171.5[142.9-214.6] | 171.5[142.9-214.5] |
|  | Trans Nzoia | 26 | 173.6[144.6-217.2] | 74.4[61.9-93.0] | 50.9[42.4-63.8] | 38.3[31.8-48.0] | 56.1[46.5-70.3] | 47.4[39.4-59.7] | 39.6[32.9-49.6] |
|  | Uasin Gishu | 27 | 105.1[87.5-131.5] | 43.1[35.8-53.9] | 28.6[23.8-35.9] | 21.2[17.6-26.6] | 33.5[27.8-41.9] | 28.1[23.3-35.2] | 19.0[15.7-23.8] |
|  | Elgeyo-Marakwet | 28 | 237.6[197.9-297.2] | 88.1[73.3-110.3] | 63.1[52.5-79.0] | 47.6[39.6-59.7] | 71.3[59.3-89.2] | 61.0[50.8-76.4] | 56.0[46.6-70.1] |
|  | Nandi | 29 | 145.6[121.2-182.1] | 59.8[49.7-74.8] | 40.6[33.8-50.9] | 30.8[25.6-38.7] | 45.2[37.6-56.6] | 38.5[32.0-48.3] | 31.0[25.8-38.9] |
|  | Baringo | 30 | 420.7[350.5-526.0] | 172.4[143.5-215.6] | 104.9[87.3-131.3] | 72.6[60.4-90.8] | 128.0[106.3-160.2] | 104.6[87.1-131.3] | 88.6[73.8-111.0] |
|  | Laikipia | 31 | 290[241.6-362.6] | 130.5[108.6-163.2] | 83.8[69.8-104.9] | 61.9[51.5-77.5] | 107.0[88.9-133.8] | 93.6[77.9-117.3] | 85.8[71.4-107.4] |
|  | Nakuru | 32 | 117.8[98.1-147.3] | 55.7[46.3-69.6] | 35.8[29.7-44.8] | 26.5[22.0-33.3] | 36.0[29.5-45.1] | 29.7[24.7-37.9] | 28.8[24.0-36.2] |
|  | Narok | 33 | 444.9[370.6-556.2] | 192.1[160.0-240.2] | 121.7[101.3-152.2] | 91.1[75.8-114] | 152.6[127.1-190.9] | 132.3[110.2-165.5] | 108.2[90.1-135.4] |
|  | Kajiado | 34 | 368.6[307.1-460.9] | 175.0[145.7-219.0] | 108.5[90.3-135.7] | 80.6[67.1-100.9] | 150.9[125.7-188.8] | 132.2[110.1-165.4] | 108.7[90.5-136.0] |
|  | Kericho | 35 | 117.7[98.0-147.2] | 48.5[40.4-60.8] | 31.7[26.3-39.7] | 23.5[19.5-29.5] | 38.9[32.4-48.8] | 33.5[27.8-41.9] | 28.0[23.3-35.1] |
|  | Bomet | 36 | 165.5[137.8-207.0] | 73.6[61.3-92.1] | 53.4[44.4-66.9] | 40.2[33.4-50.4] | 57.1[47.5-71.5] | 49.1[40.8-61.4] | 43.4[36.1-54.4] |
| **Western** | Kakamega | 37 | 92.0[76.6-115.2] | 38.6[32.1-48.4] | 25.6[21.3-32.1] | 19.0[15.7-23.8] | 27.4[22.8-34.4] | 23.4[19.4-29.3] | 16.6[13.7-20.8] |
|  | Vihiga | 38 | 56.1[46.7-70.2] | 24.0[19.9-30.1] | 16.1[13.3-20.2] | 12.1[10.0-15.2] | 17.2[14.2-21.5] | 14.4[12.0-18.2] | 12.0[9.9-15.2] |
|  | Bungoma | 39 | 134.3[111.8-168.0] | 63.6[53.0-79.2] | 45.1[37.5-56.5] | 34.0[28.2-42.6] | 66.1[53.6-82.7] | 59.1[49.2-76.1] | 52.1[43.9-66.0] |
|  | Busia | 40 | 94.9[79.0-118.8] | 39.1[32.4-48.9] | 27.9[23.1-35.0] | 20.8[17.2-26.1] | 27.8[23.1-34.8] | 23.2[19.3-29.1] | 21.4[17.8-26.9] |
| **Nyanza** | Siaya | 41 | 111.2[92.5-139.1] | 47.0[39.1-58.7] | 30.0[24.9-37.6] | 22.5[18.7-28.3] | 31.0[25.3-38.9] | 25.2[21.0-32.4] | 23.8[19.9-30.0] |
|  | Kisumu | 42 | 86.3[71.8-108.0] | 35.4[29.4-44.3] | 23.8[19.7-29.8] | 17.7[14.7-22.2] | 25.6[21.2-32.1] | 21.4[17.8-26.9] | 18.4[15.2-23.1] |
|  | Homa Bay | 43 | 110.1[91.7-137.7] | 47.2[39.2-59.0] | 32.5[27.0-40.8] | 24.3[20.1-30.4] | 35.8[29.5-44.8] | 30.2[25.1-38.3] | 27.6[23.0-34.7] |
|  | Migori | 44 | 120.3[100.2-150.5] | 50.2[41.7-62.8] | 33.4[27.7-41.8] | 25.0[20.8-31.4] | 36.9[30.7-46.2] | 31.1[25.8-39.0] | 27.8[23.1-34.9] |
|  | Kisii | 45 | 64.7[53.8-81.0] | 27.5[22.9-34.5] | 19.9[16.5-25.0] | 14.8[12.3-18.6] | 18.9[15.7-23.7] | 15.6[12.9-19.6] | 12.9[10.7-16.3] |
|  | Nyamira | 46 | 77.9[64.8-97.5] | 31.3[26.0-39.3] | 22.6[18.7-28.3] | 16.7[13.8-21.0] | 20.3[16.8-25.5] | 17.1[14.2-21.5] | 15.0[12.5-18.9] |
| **Nairobi** | Nairobi | 47 | 26.3[21.9-33.0] | 11.8[9.7-14.5] | 9.6[8.0-12.1] | 7.1[5.8-8.9] | 12.2[9.1-15.3] | 9.7[8.1-13.6] | 5.5[4.6-7.0] |
| **National** | National |  | 270.4[225.3-338.1] | 128.5[107.0-160.7] | 85.9[71.5-107.0] | 62.8[52.2-78.6] | 111.8[92.9-139.9] | 98.4[81.9-123.5] | 87.4[72.8-109.4] |

Mean travel time to the nearest urban centre in Kenya in 2019 for seven travel scenarios at county level; walking only (scenario 1), bicycling only (scenario 2), motorcycle only (scenario 3), vehicle only (scenario 4), walking followed by motorcycle transport (scenario 5), walking followed by vehicle transport (scenario 6) and walking followed by motorcycle and then vehicle transport (scenario 7). The mean speed was varied by ±20% to define an upper and lower bound of uncertainty
