# Supplementary material for: Modelling geographical accessibility to urban centres in Kenya in 2019
Source: PLoS One. 2021 May 14;16(5):e0251624. doi: 10.1371/journal.pone.0251624 (PMC8127925; doi:10.1371/journal.pone.0251624)
Supplement: S3 Appendix — (DOCX) [file pone.0251624.s003.docx]

**S3 Appendix**

The proportion of population within 2-hours travel time of the nearest urban centre in Kenya in 2019 for 7 travel scenarios; walking only (scenario 1), bicycling only (scenario 2), motorcycle only (scenario 3), vehicle only (scenario 4), walking followed by motorcycle transport (scenario 5), walking followed by vehicle transport (scenario 6) and walking followed by a motorcycle and then vehicle transport (scenario 7). The mean speed was varied by ±20% to define an upper and lower bound of uncertainty.

| **Province** | **County** | **ID** | **Scenario 1** | **Scenario 2** | **Scenario 3** | **Scenario 4** | **Scenario 5** | **Scenario 6** | **Scenario 7** |
| --- | --- | --- | --- | --- | --- | --- | --- | --- | --- |
| Coast | Mombasa | 1 | 99.52[99.43-99.5] | 99.58[99.53-99.58] | 94.17[94.13-94.17] | 94.17[94.13-94.17] | 99.57[99.57-99.57] | 99.57[99.57-99.57] | 99.54[99.54-99.54] |
|  | Kwale | 2 | 52.26[42.02-60.52] | 90.51[81.24-95.07] | 98.12[96.71-98.64] | 98.8[98.24-99.11] | 94.67[91.49-95.64] | 95.15[93.4-96.08] | 97.94[97.36-98.34] |
|  | Kilifi | 3 | 63.94[55.64-69.25] | 88.42[84.15-91.51] | 94.75[91.15-95.83] | 96.15[95.29-96.42] | 95.26[92.56-96.79] | 96.74[94.94-97.29] | 97.62[96.85-97.87] |
|  | Tana River | 4 | 26.94[22.92-31.19] | 44.3[39.07-50.75] | 66.98[57.01-71.98] | 76.59[71.33-79.37] | 53.54[47.43-58] | 57.19[52.39-61.06] | 57.41[52.75-61.19] |
|  | Lamu | 5 | 58.74[55.79-62.14] | 71.28[67.27-74.59] | 72.32[70.65-73.39] | 74[72.85-75.21] | 70.2[67.61-72.41] | 70.79[68.28-73.16] | 71.57[70.07-73.39] |
|  | Taita Taveta | 6 | 62.54[53.75-72.1] | 95.61[91.1-97.42] | 99.65[99.07-100] | 100[99.88-100] | 97.59[95.72-98.16] | 97.94[96.73-98.51] | 98.58[97.74-99.15] |
| North Eastern | Garissa | 7 | 33.5[31.72-35.02] | 39.37[37.18-41.79] | 45.87[42.49-49.23] | 53.57[48.76-61.96] | 41.49[38.35-44.28] | 44.85[40.66-50.59] | 46.38[42.53-52.32] |
|  | Wajir | 8 | 18.68[17.27-20.12] | 27.83[24.22-31.81] | 41.38[34.01-48.74] | 55.94[45.94-65.62] | 33.03[27.92-38.03] | 37.82[32.11-43.89] | 38.06[32.29-44.24] |
|  | Mandera | 9 | 31.91[29.65-34.59] | 46.66[40.05-52.93] | 66.98[56.24-77.15] | 85.21[74.09-91.76] | 56.19[47.49-63.95] | 63.45[55-70.71] | 63.45[55.01-70.69] |
| Eastern | Marsabit | 10 | 33.69[31.34-36] | 46.73[42.16-50.7] | 59.55[53.44-65.81] | 70.11[62.32-75.99] | 51.16[46.62-55.8] | 55.59[50.25-60.18] | 55.59[50.26-60.18] |
|  | Isiolo | 11 | 52.21[49.02-54.45] | 62.83[58.73-67.06] | 70.56[64.35-75.52] | 78.75[72.18-84.97] | 67.13[63.06-72.62] | 71.2[65.53-78.34] | 71.42[65.77-78.45] |
|  | Meru | 12 | 66.48[55.9-73.29] | 99.47[96.38-99.9] | 99.4[99.27-99.43] | 99.43[99.42-99.43] | 99.23[97.72-99.75] | 99.42[98.31-99.86] | 99.71[99.22-99.9] |
|  | Tharaka-Nithi | 13 | 55.71[45.83-63.40] | 90.29[79.42-97.16] | 100[100-100] | 100[100-100] | 95.38[90.63-97.52] | 96.78[93.35-97.92] | 98.22[97.39-98.53] |
|  | Embu | 14 | 59.75[47.34-69.02] | 90.38[84.09-94.78] | 99.08[96.54-99.45] | 99.46[99.34-99.46] | 97.76[94.16-99.01] | 99.05[97.34-99.39] | 99.6[99.39-99.58] |
|  | Kitui | 15 | 20.34[15.98-24.97] | 48.54[41.11-55.11] | 73.4[61.4-85.05] | 93.28[82.4-97.09] | 68.95[57.41-79.09] | 82.34[72.35-88.88] | 92.31[82.16-96.47] |
|  | Machakos | 16 | 68.53[59.52-76.16] | 92.86[89.41-96.06] | 99.52[98.18-99.61] | 99.62[99.58-99.62] | 98.09[94.92-98.97] | 98.95[97.24-99.05] | 99.09[98.96-99.07] |
|  | Makueni | 17 | 34.67[24.84-46.26] | 85.58[75.01-92.09] | 97.36[91.61-99.51] | 99.62[98.12-99.64] | 88.86[82.89-93.09] | 91.64[86.94-95.02] | 98.15[96.83-98.61] |
| Central | Nyandarua | 18 | 76.43[63.11-84.67] | 99.9[98.21-99.91] | 99.94[99.94-99.95] | 99.95[99.95-99.95] | 99.67[98.99-99.86] | 99.82[99.26-99.86] | 99.89[99.72-99.95] |
|  | Nyeri | 19 | 90.45[80.99-93.59] | 99.73[98.77-99.8] | 100[100-100] | 100[100-100] | 99.67[99.58-99.73] | 99.7[99.59-99.7] | 99.77[99.65-99.76] |
|  | Kirinyaga | 20 | 98.17[91.87-99.70] | 100[100-100] | 99.93[99.93-99.93] | 99.93[99.93-99.93] | 100[100-100] | 100[100-100] | 100[100-100] |
|  | Murang'a | 21 | 72.06[55.44-83.67] | 99.89[98.63-99.95] | 100[100-100] | 100[100-100] | 99.74[99.35-99.95] | 99.94[99.56-99.95] | 99.95[99.95-99.95] |
|  | Kiambu | 22 | 95.07[91.54-97.57] | 99.94[99.89-99.94] | 100[100-100] | 100[100-100] | 99.93[99.91-99.94] | 99.96[99.93-99.94] | 99.96[99.93-99.94] |
| Rift Valley | Turkana | 23 | 22.16[20.57-23.66] | 30[26.65-34.06] | 47.58[39.24-56.95] | 63.39[53.02-71.34] | 36.16[30.9-41.8] | 42.4[36.02-47.14] | 42.4[36.02-47.14] |
|  | West Pokot | 24 | 21.44[17.94-23.99] | 52.77[42.09-62.04] | 74.47[62.44-81.34] | 84.78[79.42-86.54] | 62.25[53.24-68.35] | 67.49[60.43-72.49] | 68.08[61.67-73] |
|  | Samburu | 25 | 22.44[17.44-28.88] | 50.42[41.35-59.9] | 74.65[62.48-82.68] | 87.34[78.6-93.75] | 62.95[52.12-70.41] | 68.49[59.16-75] | 68.49[59.16-75] |
|  | Trans Nzoia | 26 | 51.49[39.66-63.67] | 95.95[89.14-98.87] | 100[100-100] | 100[100-100] | 99.44[98.66-99.59] | 99.54[99.28-99.67] | 99.66[99.49-99.77] |
|  | Uasin Gishu | 27 | 74.95[67.22-81.53] | 99.44[96.71-99.78] | 98.81[98.8-98.81] | 98.81[98.8-98.81] | 99.64[99.27-99.76] | 99.66[99.54-99.76] | 99.79[99.75-99.78] |
|  | Elgeyo-Marakwet | 28 | 28.23[22.14-36.44] | 85.2[70.7-92.42] | 100[92.66-100] | 100[100-100] | 94.86[88.88-97.68] | 96.41[92.2-98.55] | 97.36[94.29-98.82] |
|  | Nandi | 29 | 49.39[39.90-60.20] | 99.33[94.14-100] | 99.54[99.45-99.54] | 99.54[99.54-99.54] | 99.76[98.51-99.95] | 99.91[99.05-99.97] | 99.98[99.56-100] |
|  | Baringo | 30 | 28.86[24.22-33.00] | 63.24[52.58-70.35] | 81.23[73.57-87.82] | 94.36[86.9-97.7] | 75.98[67.53-82.6] | 83.83[76.81-87.74] | 87.49[81.25-91.06] |
|  | Laikipia | 31 | 57.03[50.12-64.14] | 91.76[86.63-94.08] | 98.32[95.97-99.1] | 99.32[98.73-99.63] | 93.31[89.3-95.9] | 94.87[91.42-96.81] | 96.77[94.99-97.81] |
|  | Nakuru | 32 | 79.37[71.99-84.25] | 96.93[93.44-99.01] | 99.9[99.61-99.91] | 99.91[99.91-99.91] | 99.57[98.93-99.73] | 99.68[99.43-99.75] | 99.72[99.49-99.77] |
|  | Narok | 33 | 27.44[21.35-33.22] | 63.74[50.76-74] | 82.81[73.63-86.98] | 88.73[84.22-91.81] | 72.02[61.76-79.82] | 77.64[68.37-84.28] | 87.14[80.61-91.47] |
|  | Kajiado | 34 | 56.71[51.55-60.75] | 75.43[69.28-81.28] | 90.05[84.06-93] | 94.27[91.02-96.24] | 81.44[75.02-86.12] | 85.11[79.33-89.29] | 90.4[85.67-93.38] |
|  | Kericho | 35 | 64.55[51.8-75.64] | 99.79[97.91-100] | 98.65[98.59-98.65] | 98.65[98.64-98.65] | 99.92[98.92-100] | 99.95[99.51-100] | 99.97[99.68-100] |
|  | Bomet | 36 | 52.27[38.06-67.43] | 95.96[89.5-99.46] | 99.8[99.79-99.8] | 99.8[99.8-99.8] | 99.54[99.36-99.54] | 99.57[99.49-99.54] | 99.57[99.53-99.54] |
| Western | Kakamega | 37 | 78.3[65.16-88.41] | 99.31[99-99.31] | 100[100-100] | 100[100-100] | 99.31[99.26-99.31] | 99.32[99.29-99.31] | 99.31[99.31-99.31] |
|  | Vihiga | 38 | 98.54[94.47-99.55] | 100[100-100] | 100[100-100] | 100[100-100] | 100[99.99-100] | 99.99[99.99-100] | 99.99[99.99-100] |
|  | Bungoma | 39 | 75.01[60.22-86.34] | 99.83[99.56-99.84] | 100[100-100] | 100[100-100] | 99.83[99.8-99.83] | 99.82[99.81-99.83] | 99.83[99.82-99.84] |
|  | Busia | 40 | 71.3[55.29-84.96] | 99.17[99.2-99.23] | 100[100-100] | 100[100-100] | 99.23[99.02-99.16] | 99.23[99.08-99.16] | 99.25[99.08-99.16] |
| Nyanza | Siaya | 41 | 64.84[51.92-75.07] | 93.49[90.97-95.43] | 95.68[95.65-95.68] | 95.68[95.67-95.69] | 96.67[96.13-96.69] | 97.87[96.63-96.69] | 97.87[96.64-96.72] |
|  | Kisumu | 42 | 84.96[78.34-90.15] | 99.85[99.01-99.82] | 98.94[98.94-98.94] | 98.94[98.94-98.94] | 99.79[99.74-99.83] | 99.86[99.77-99.83] | 99.83[99.83-99.83] |
|  | Homa Bay | 43 | 59.09[46.62-73.73] | 96.63[95.78-96.75] | 99.38[99.37-99.38] | 99.38[99.37-99.38] | 96.22[94.93-96.62] | 99.34[95.5-96.7] | 99.63[95.98-96.76] |
|  | Migori | 44 | 71.67[58.84-79.49] | 98.53[93.8-99.91] | 100[100-100] | 100[100-100] | 99.84[98.8-99.84] | 99.91[99.53-99.84] | 99.91[99.78-99.84] |
|  | Kisii | 45 | 90.57[78.29-98.53] | 100[100-100] | 100[100-100] | 100[100-100] | 100[100-100] | 100[100-100] | 100[100-100] |
|  | Nyamira | 46 | 85.94[64.06-98.05] | 99.98[99.98-99.98] | 97.54[97.54-97.54] | 97.54[97.54-97.54] | 99.98[99.98-99.98] | 99.98[99.98-99.98] | 99.98[99.98-99.98] |
| Nairobi | Nairobi | 47 | 99.91[99.87-99.93] | 99.93[99.93-99.93] | 99.5[99.5-99.5] | 99.5[99.5-99.5] | 99.93[99.93-99.93] | 99.93[99.93-99.93] | 99.99[99.99-99.99] |
| National | National |  | 66.44[58.59-72.72] | 87.9[84.48-90.11] | 92.96[90.79-94.44] | 95.46[93.89-96.51] | 90.77[88.47-92.35] | 92.33[90.31-93.56] | 93.32[91.73-94.28] |
