# Supplementary material for: Modelling geographical accessibility to urban centres in Kenya in 2019
Source: PLoS One. 2021 May 14;16(5):e0251624. doi: 10.1371/journal.pone.0251624 (PMC8127925; doi:10.1371/journal.pone.0251624)

**S4 Appendix**

Correlation matrix between travel time to the nearest urban centre for the most pragmatic scenario (7) and ten SDG indicators. The coefficients between the SDG indicators is also presented. The definitions of the indicators are included in Table 2 in the main manuscript


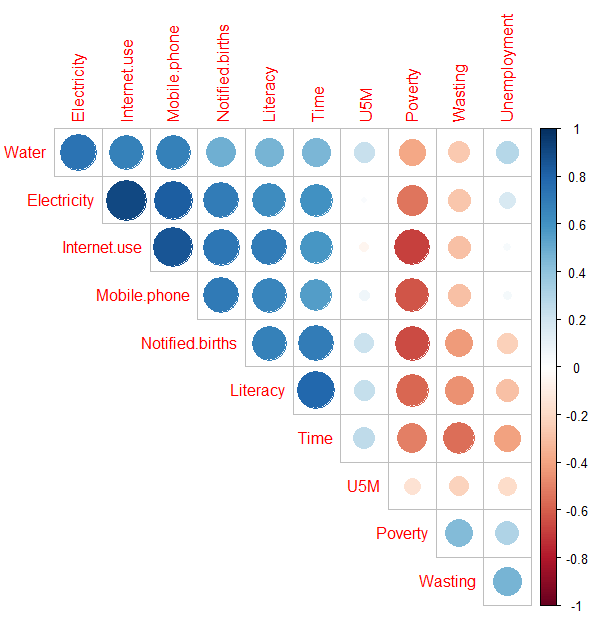

Supplement: S4 Appendix — (DOCX) [file pone.0251624.s004.docx]
